# Free fatty acid can induce cardiac dysfunction and alter insulin signaling pathways in the heart

**DOI:** 10.1186/s12944-018-0834-1

**Published:** 2018-08-08

**Authors:** Lina Han, Jiali Liu, Leilei Zhu, Fang Tan, Yupei Qin, He Huang, Yerong Yu

**Affiliations:** 1 Department of endocrinology and metabolism, West China Hospital, Sichuan University, Guoxue lane 37, Chengdu City, Sichuan Province 610041 People’s Republic of China; 2 Department of Cardiovascular, West China Hospital, Sichuan University, Chengdu City, Sichuan Province People’s Republic of China

**Keywords:** Free fatty acids, Hyperinsulinemia, Insulin resistance, Cardiac dysfunction

## Abstract

**Background:**

Insulin resistance has been independently related to heart failure. However, the specific mechanisms of high FFA levels in the pathophysiology of heart failure in insulin-resistant states are remain largely unclear. This study investigated whether elevated circulating free fatty acids (FFA) levels result in impaired cardiac structure and function in vivo via insulin-related signaling pathways in myocardium.

**Methods:**

Male Wistar rats were randomly divided into the intralipid group (20% intralipid plus heparin infusion) and the control group (glycerol infusion). Blood samples were collected before and after 6-, 12-, and 24-h infusions. Cardiac structure and function were measured using echocardiography. Maximum velocity of myocardial contraction (+dP/dt _max_) and diastole (−dP/dt _max_) were measured using a physiological polygraph in vivo. Heart tissues were collected for western blotting.

**Results:**

Compared with the control group, plasma FFA, plasma glucose, and serum insulin levels increased significantly in the intralipid group. With increasing infusion time, cardiac function in the intralipid group decreased gradually compared with the control group. After a 24-h infusion, early (E’, cm/s) diastolic peak velocities and (−dP/dt _max_) decreased significantly. Protein expression of phosphatidylinositol 3-kinase (PI3K), the serine/threonine kinase Akt, and phosphorylated Akt in myocardium increased after a 6-h infusion and decreased significantly after a 24-h infusion in the intralipid group. Protein expression of glucose transporter type 4 (GLUT4), Adenosine 5′-monophosphate -activated protein kinase (AMPK), phosphorylated AMPK(p-AMPK), and endothelial nitric oxide synthase (eNOS) in myocardium gradually decreased in the intralipid group.

**Conclusions:**

Elevated FFA levels may impair cardiac function and cardiac dysfunction might result from myocardial insulin resistance with significant changes to PI3K-Akt-GLUT4 and AMPK-eNOS signaling pathways with increasing FFA levels.

## Background

Heart failure is a leading cause of morbidity and mortality globally. Insulin resistance is a major risk factor for heart failure [1–3] and plays an important role in the development of left ventricular (LV) dysfunction [2, 3]. Hyperinsulinemia insulin-resistant states could also contribute to cardiac remodeling and ventricular dysfunction through the growth-promoting activity of insulin [4].

The cardiomyocyte is a typical insulin-targeted cell. In a healthy heart, insulin activates phosphatidylinositol 3-kinase (PI3K) -Akt signaling and promotes glucose transporter type 4 (GLUT4) translocation to the cell surface to facilitate glucose transport [5]. The failure of insulin-stimulated translocation of GLUT4 to the plasma membrane is an early step in the development of insulin resistance [6]. Studies suggest that in insulin-resistant cardiomyopathy, reduced myocardial glucose uptake [7] and damaged LV structure and function [8] are associated with alterations in Akt activity and impaired GLUT4 translocation [9, 10].

Endothelial nitric oxide synthase (eNOS) can directly modify insulin-dependent cardiac molecular pathways involved in the control of coronary tone and coronary circulatory function [11]. However, insulin resistance can impair the activity of eNOS, reduce nitric oxide (NO) generation, which may subsequently reduce peripheral perfusion, limit the blood flow provided by the supplying large vessels, increase impedance in the failing LV, and consequently impair LV function [12].

An increased plasma free fatty acid (FFA) concentration is a typical metabolic abnormality in insulin-resistant states [13]. Acute increases in plasma FFA levels can induce insulin resistance by inhibiting glucose uptake in skeletal muscle, hepatocytes, and adipocytes, and by attenuating insulin signaling in insulin receptor substrate 1 (IRS-1) -PI3K activity [14]. Kim et al. reported that fatty acids as a nutrient stress activates cardiac inflammation and suppresses myocardial glucose metabolism via inhibition of AMPK and IRS-1 in the heart [15]. Additionally, we previously reported that high-fat diet-induced cardiac dysfunction could be improved by reducing FFA levels [16]. The molecular mechanisms of high FFA levels in changed insulin signaling in cardiomyocytes and the pathophysiology of heart failure in insulin-resistant states are incompletely understood.

In the current study, we investigated the effects of elevated levels of circulating FFA in vivo on cardiac structure and function via changed PI3K-Akt and AMPK signaling pathways in the heart. Male Wistar rats were used in which plasma FFA concentrations were acutely raised using intralipid plus heparin infusion. Infused rats were evaluated for cardiac structure and function. Rat LV tissue was also evaluated for protein expression of PI3K, Akt, p-Akt, GLUT4, AMPK, p-AMPK, and eNOS.

## Methods

### Animal experiments

Eight-week-old male Wistar rats (weighing 300–400 g) were obtained from the Animal Resources Centre (Sichuan University, Chengdu, China) and housed in environmentally controlled conditions at 22 °C on a 12-h light/dark cycle and with free access to food. Rats were fed with standard chow for 1 week. Rats were catheterized under pentobarbital anesthesia (PE-50, Cay Adams, Boston, MA) with tubing inserted into the left carotid artery for sampling, and SILASTIC tubing inserted into the right jugular vein for infusion, as described previously [17]. Rats were left to recover over the following 5–7 days until their weights were stable and were studied while awake and unstressed after an overnight fast. Rats were randomly divided into two groups: experimental group (intralipid group, which underwent infusion with 20% intralipid (Sino-Swed Pharmaceutical Corp. Ltd., Beijing, China) and heparin (Nanjingxinbai Pharmaceutical Co, Jiangsu Sheng, China) at a rate of 0.6 ml/h (*n* = 30), and the control group (glycerol group, which underwent infusion with 2.5% glycerol (Biosharp Corp. Ltd., Shenzhen, China) at the same rate (*n* = 30). All experimental procedures were approved by the Animal Experimentation Ethics Committee (West China Hospital, Sichuan University) and were in accordance with the Guide for Care and Use of Laboratory Animals of the National Institutes of Health.

### Study protocol

Blood was collected before infusion and after 6-, 12-, and 24-h infusions. After infusions, rats underwent pentobarbital sodium anesthesia and cardiac structure and function were measured using echocardiography and invasive hemodynamic determination. Plasma FFA, plasma glucose, and serum insulin levels were measured. Histology and morphology of heart tissues were observed in samples stained with picrosirius red. Protein expression of PI3K, Akt, p-Akt, GLUT4, eNOS, AMPK, and p-AMPK in myocardium were determined using western blotting.

### Cadiac function

Following infusion, echocardiography was performed to assess cardiac structure and function. Rats were anaesthetized and placed on the micromanipulator stage. M-mode echocardiography was performed to measure (in mm) LV inner dimension-diastole (LVIDd), LV inner dimension-systole (LVIDs), interventricular septum thickness (IVSd), left ventricular end-diastolic posterior wall thickness (LYPWT), LV ejection fraction (EF %) and fractional shortening (FS %), and peak early (E, cm/s) and late (A, cm/s) flow velocities, early (E’, cm/s) and late (A’, cm/s) diastolic peak velocities. Three cardiac cycles were recorded and the means of the above indices from the three cardiac cycles were computed.

Following echocardiographic examination, the right common carotid artery was isolated and cannulated using a pressure transducer (PowerLab; AD Instruments, Sydney, Australia). The catheter was advanced to the aorta, passed through the aortic valve, and stabilized in the LV cavity. Maximum rise and drop in the speed of internal pressure in the LV (+dP/dt _max_ and − dP/dt _max_) were measured by physiological polygraph to evaluate cardiac function, as described previously [16].

### Biochemical analysis

Plasma glucose levels were measured using the glucose oxidase method. Plasma insulin levels were measured using ELISA (EMD Millipore, Billerica, MA). FFA levels were assayed using the colorimetric method with a serum FFA kit (E1001; Applygen Technologies, Beijing, China).

### Western blotting

For the western blot analysis, 50 mg of LV myocardial tissue was homogenized on ice in radioimmunoprecipitation lysis buffer containing a complete protease inhibitor cocktail (Roche Diagnostics). The homogenates were centrifuged to collect the supernatants. The protein concentration was determined using a BCA protein assay kit (Thermo Scientific). LV myocardial samples were subjected to SDS-PAGE and transferred to polyvinylidene difluoride membranes. Membranes were blocked with 5% BSA in PBS plus Tween 20 at room temperature for 2 h, and then incubated with anti-PI3K (P110α), anti-Akt (Ser172), anti-phospho-Akt (Ser172), anti-GLUT4, anti-AMPK (Thr172), anti-phospho-AMPK (Thr172), and anti-eNOS antibodies (Cell Signaling Technology, Danvers, MA) at 4 °C overnight. Membranes were subsequently washed and incubated with the relevant secondary horseradish peroxidase-conjugated antibodies (1:5000; ZSGB-BIO, Beijing, China) for 90 min. β-actin (mouse anti-β-actin; 1:500; ZSGB-BIO) was used as the loading control. Intensity was measured using Quantity One densitometric software (Bio-Rad Laboratories, Hercules, CA), as described previously [18].

### Statistical analysis

Data were analyzed using SPSS 22.0. Results are expressed as mean ± SEM. Student’s *t*-test was used to compare measures between control and experimental groups. ANOVA was used to compare means of multiple groups. A value of *P* < 0.05 was considered statistically significant.

## Results

### Biometric parameters

There were no statistically significant differences in body weights of rats before surgery (B-S) and before infusion (B-I), and in circulating levels of FFA, glucose, and insulin between the two groups at the beginning of the study (*p* > 0.05) (Table 1).

**Table 1:** Biometric and blood parameters of rats at basal condition in two groups

	Control	Intralipid
B-S weight, (g)	383.05 ± 2.31	382.95 ± 2.56
B-I weight, (g)	373.03 ± 2.41	374.12 ± 2.42
FFA, (μmol/l)	339.96 ± 9.08	323.85 ± 8.92
Fasting glucose, (mmol/l)	5.37 ± 0.08	5.27 ± 0.08
Fasting insulin, (ng/ml)	0.41 ± 0.01	0.43 ± 0.02

Data are expressed as mean ± SEM. B-S weight, body weights of rats before surgery; B-I, body weights of rats before infusion. *p* > 0.05

### LV structure and function

After infusing for 6, 12, or 24 h, there were no significant differences in heart structure (LVIDs, LVIDd, ISVD, LYPWT), FS %, and EF % in the studied groups (*p* > 0.05) (Table 2). With increasing infusion time, cardiac function (E, A, E’, A’, +dP/dt _max_, −dP/dt _max_) in the intralipid group decreased gradually compared with the control group. After intravenous infusion for 24 h, E’ and − dP/dt _max_ decreased significantly (*p* < 0.05) (Table 2).

**Table 2:** Left ventricular structure and function in the studied groups

	Control			Intralipid		
Time(h)	6 h	12 h	24 h	6 h	12 h	24 h
LVIDs, (mm)	3.32 ± 0.05	3.10 ± 0.20	3.30 ± 0.25	3.15 ± 0.23	3.33 ± 0.20	3.40 ± 0.17
LVIDd, (mm)	5.73 ± 0.25	5.77 ± 0.35	5.73 ± 0.24	5.69 ± 0.23	5.62 ± 0.19	5.64 ± 0.32
ISVD, (mm)	1.64 ± 0.11	1.59 ± 0.06	1.73 ± 0.07	1.71 ± 0.16	1.58 ± 0.08	1.75 ± 0.09
LYPWT, (mm)	1.67 ± 0.07	1.67 ± 0.08	1.74 ± 0.06	1.81 ± 0.08	1.67 ± 0.05	1.95 ± 0.13
FS, (%)	43.65 ± 1.91	43.90 ± 2.41	38.91 ± 2.29	40.93 ± 2.02	37.33 ± 2.43	41.55 ± 1.86
EF, (%)	80.63 ± 2.02	80.58 ± 2.53	75.05 ± 2.67	77.60 ± 2.01	78.10 ± 1.94	73.38 ± 3.05
E, (cm/s)	107.00 ± 4.88	106.3 ± 4.72	105.00 ± 6.55	104.30 ± 5.28	102.50 ± 4.57	89.500 ± 4.11
A, (cm/s)	89.00 ± 4.18	80.00 ± 4.83	77.25 ± 5.41	87.50 ± 4.44	73.25 ± 3.28	68.50 ± 6.12
E’, (cm/s)	8.50 ± 0.65	7.88 ± 0.43	8.00 ± 0.41	8.00 ± 0.41	7.50 ± 0.65	5.63 ± 0.43^*^
A’, (cm/s)	6.63 ± 0.38	6.37 ± 0.55	6.50 ± 0.46	6.00 ± 0.61	5.13 ± 0.63	4.13 ± 0.66
+dP/dt _max_	5285 ± 101.8	5238 ± 142.2	5160 ± 68.32	5136 ± 216.0	4614 ± 149.3	4336 ± 137.0
-dP/dt _max_	4858 ± 79.30	4680 ± 123.1	4651 ± 159.5	4760 ± 127.1	4472 ± 139.9	3665 ± 161.1^*^

Data are expressed as mean ± SEM. Control, control group; Intralipid, intralipid group. ^*^p < 0.05 vs the control group

### Serum biochemical analysis

Circulating FFA levels increased and levels of glucose and insulin gradually increased following intralipid plus heparin infusion for 6–24 h in rats (*p* < 0.001) (Table 3). Following infusion for 6–24 h, plasma FFA levels increased 1.58–2.76-fold compared with the glycerol-infusion control group. Compared with the control group, serum glucose and insulin levels in the intralipid group increased 1.37-fold and 1.79-fold, respectively, after a 6-h infusion, increased 1.52-fold and 2.14-fold, respectively, after a 12-h infusion, and increased 1.69-fold and 2.41-fold, respectively, after a 24-h infusion (*p* < 0.001). The homeostasis model assessment (HOMA) index for insulin resistance (HOMA-IR) values also increased gradually during intralipid infusion (6 h: 5.78, 12 h: 7.55, 24 h: 9.36).

**Table 3:** Values of serum FFA, glucose and insulin in the studied groups

Time (h)	6 h	12 h	24 h
FFA, (μmol/l)			
Control	349.89.2 ± 23.42	356.15 ± 23.00	342.81 ± 15.10
Intralipid	836.51 ± 34.13^***^	1029.84 ± 28.52^***^	1218.80 ± 64.14^***^
Glucose, (mmol/l)			
Control	5.95 ± 0.17	6.00 ± 0.18	5.93 ± 0.31
Intralipid	8.18 ± 0.22^***^	9.1 ± 0.35^***^	10.03 ± 0.41^***^
Insulin, (ng/ml)			
Control	0.42 ± 0.01	0.41 ± 0.02	0.41 ± 0.01
Intralipid	0.75 ± 0.02^***^	0.88 ± 0.02^***^	0.99 ± 0.04^***^

Data are expressed as mean ± SEM. Control, control group; Intralipid, intralipid group. ^***^p < 0.001 vs the control group

### Changes in protein expression of PI3K, Akt, p-Akt, and GLUT4 in LV myocardial tissue

**Fig. 1:**
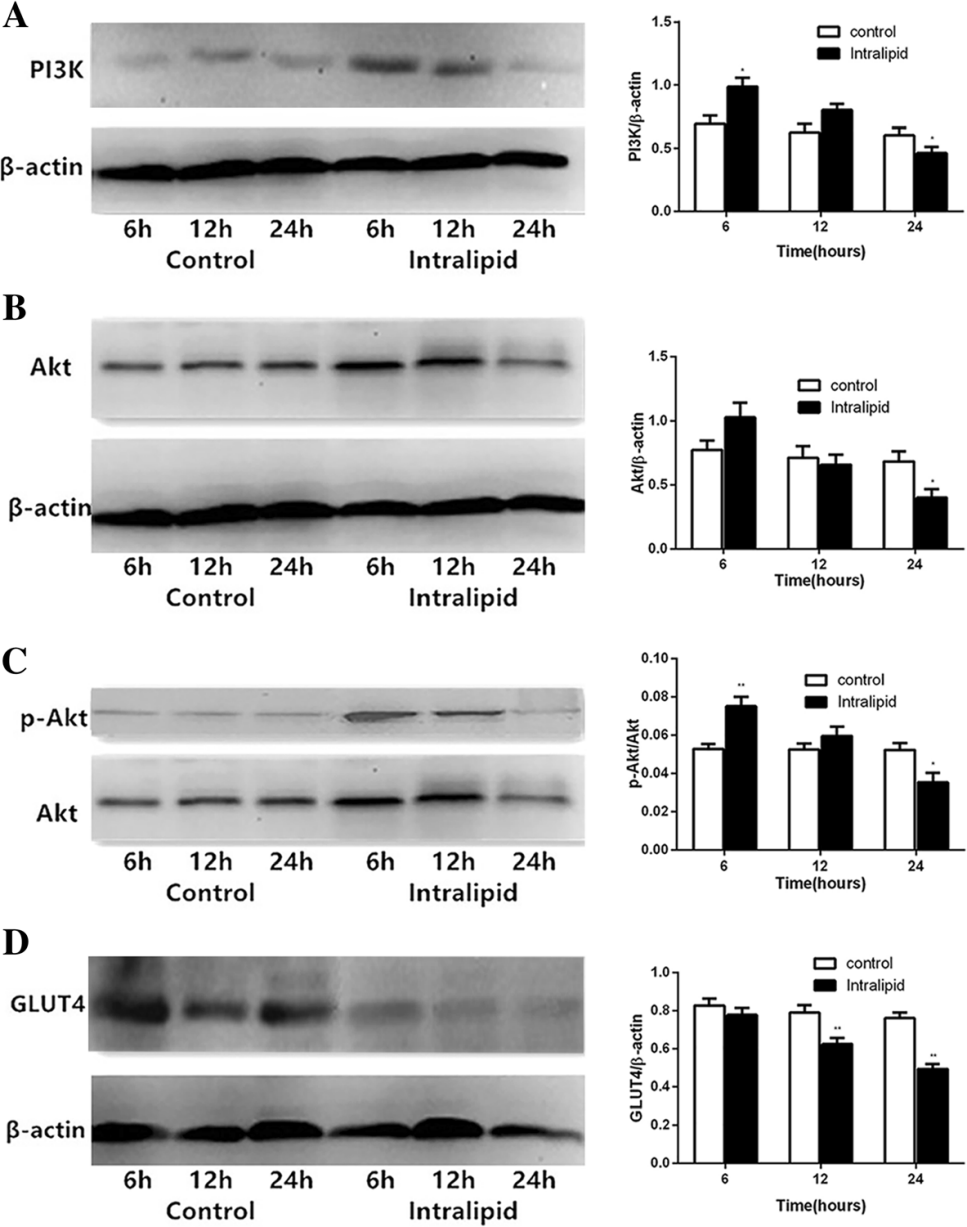
Changes in expression levels of PI3K, Akt, p-Akt, and GLUT4 protein in LV myocardial tissue in rats. Data are shown as mean ± SEM (*n* = 5–7). ^*^p < 0.05, vs the control group. ^**^p < 0.01, vs the control group. Control: glycerol control group; Intralipid: intralipid and heparin infusion group

No statistically significant differences in protein expression levels of PI3K, Akt, p-Akt, and GLUT4 were observed in the control group over the 6–24 h glycerol infusion with western blotting (*p* > 0.05). PI3K, Akt, and p-Akt in the intralipid group increased by 41.64% (*p* < 0.05), 34.02 (*p* > 0.05), and 28.83% (*p* < 0.05), respectively, at 6 h, and subsequently declined but remained at similar levels as the control group at 12 h (*p* > 0.05), and then dramatically decreased by 33.41% (*p* < 0.05), 41.30% (*p* < 0.05), and 28.33% (*p* < 0.05) at 24 h (Fig. 1). GLUT4 protein levels at 12 and 24 h decreased by 20.96% (*p* < 0.01) and 35.03% (*p* < 0.01), respectively. There was no difference in GLUT4 protein levels at 6 h in the intralipid group compared with the control group (Fig. 1). These temporary changes in the expression of PI3K, Akt, p-Akt, and GLUT4 protein after lipid infusion in this model are consistent with insulin signaling changes at different stages of heart failure.

### FFA inhibited AMPK–eNOS signaling pathway activities

**Fig. 2:**
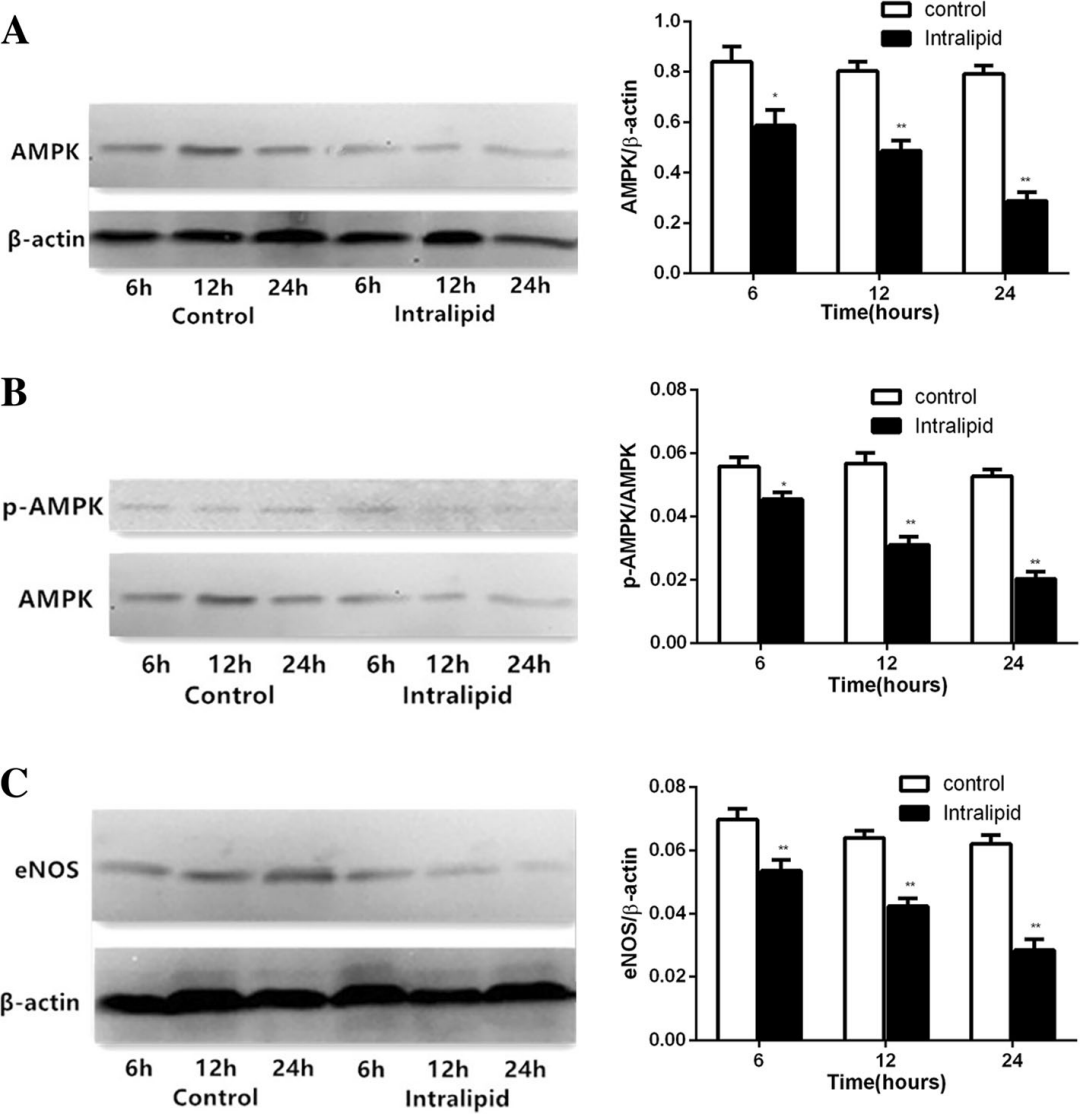
Changes in expression levels of AMPK, p-AMPK, eNOS protein in LV myocardium. Data are shown as mean ± SEM (*n* = 5–7). ^*^p < 0.05, vs the control group; ^**^p < 0.01, vs the control group. Control: glycerol control group; Intralipid: intralipid and heparin infusion group

Total AMPK, p-AMPK, and eNOS protein levels did not significantly change in the control group during glycerol infusion for 6–24 h. During intralipid infusion, total AMPK, p-AMPK, and eNOS protein levels gradually but markedly deceased by 30.08% (*p* < 0.05), 39.42% (*p* < 0.05), and 63.64% (*p* < 0.01), respectively, at 6 h, by 18.51% (*p* < 0.01), 45.42% (*p* < 0.01), and 61.56% (*p* < 0.01), respectively, at 12 h, and by 23.24% (*p* < 0.01), 33.74% (*p* < 0.01), and 54.11% (*p* < 0.01), respectively, at 24 h (Fig. 2). These results are consistent with the effects of high FFA on the regulation of cardiovascular AMPK and eNOS.

## Discussion

In this study, we observed that intravenous lipid and heparin infusion-induced elevation of circulating FFA levels could impair cardiac structure and function in rats. It is suggested that the molecular mechanisms responsible for this damaging effect on the heart were via altering PI3K–Akt–GLUT4 signaling pathways and reducing AMPK–eNOS signaling pathways in myocardium. Over the 6–24-h infusion period, serum FFA levels in the intralipid group increased 1.58–2.76-fold compared with the control group. These findings are similar to previous reports [17, 19].

We previously reported that elevated circulating FFA levels in rats can acutely increase insulin release from islets of β-cells [17]. In the current study, we observed that the concentration of insulin and glucose rapidly and gradually increased over time, and that the ratio of insulin-to-glucose also gradually increased (6 h: 15.79, 12 h: 16.65, 24 h: 17.00) in the lipid infusion study. These results suggest that the response of peripheral tissues to insulin decreased in the intralipid group, and the effects of high levels of FFA on glucose-induced insulin secretion appeared time-dependent.

Previous study [20] have reported that muscle glycogen synthesis rates were similar to the control group during a 3-h intralipid infusion, but subsequently decreased to approximately 50% of control values after 6 h of intralipid infusion. Furthermore, studies report that short-term lipid infusion stimulates, whereas long-term infusion inhibits, glucose-induced insulin secretion, suggesting time dependency of the effects of intralipid infusion on glucose-induced insulin secretion [21, 22].

As the infusion time increased in the current study, cardiac function (E, A, E’, A’, +dP/dt _max_, −dP/dt _max_) in the intralipid group decreased gradually. After 24 h of lipid infusion, diastolic function, including E’ and LV − dP/dt _max_, significantly decreased compared with the control group. We hypothesized that high FFA levels may reduce cardiac function. There were no significant differences in heart structure, FS %, and EF % between groups over the 24-h infusion period. Many previous studies that have reported the results of LV hypertrophy and remodeling were conducted using an animal model of obesity, which is regarded as a chronic disease, whereas in the current study, the rat model used acute elevation in circulating FFA.

It is reported that short-term lipid infusion reduces both glucose oxidation and glycogen synthesis and is associated with impaired glucose transport. The mechanism underlying this reduction is suggested to be that increased FFA results in decreased insulin stimulation of tyrosine phosphorylation of IRS-1 and down-regulated IRS-1-associated PI3K activity, which impair insulin-stimulated phosphorylation of Akt and GLUT4 translocation [23–25]. Although animal and human studies have shown that elevated circulating FFA can directly induce insulin resistance in some tissues such as muscles, adipose tissues, the liver, and endothelial tissues, the exact mechanism by which FFA changes insulin signaling in the heart is not completely understood.

In the current study, we found that PI3K, Akt, and p-Akt levels in the myocardium increased after the first 6 h of intralipid infusion, tended to decrease after 12 h of intralipid infusion, and then markedly decreased after 24 h of intralipid infusion. Although 6 h of lipid infusion did no change the GLUT4 protein level, GLUT4 remarkably decreased after 12 and 24 h of lipid infusion compared with the control group. Overall, we observed that 6 h of lipid infusion up-regulated myocardial insulin sensitivity, whereas 24 h of lipid infusion significantly reversed it. Such a finding suggests that myocardial insulin resistance progressed during intralipid infusion over time.

The reason for the increased insulin sensitivity after 6 h of lipid infusion might be because the heart is different from other tissues. It is suggested that the heart has developed sophisticated systems to detect and respond to nutrient overload in insulin-resistant states [26]. Recent studies on insulin signaling in the heart in animal models of insulin resistance have reported varying and potentially divergent results, possibly reflecting the different models and experimental conditions [27]. Insulin-mediated activation of Akt increases in 45% fat diet-induced obesity animal models [28], whereas activation is preserved or decreases in more severe insulin resistance models such as 60% fat diet-induced obesity animals or ob/ob mice [29]. Furthermore, the molecular mechanisms linking increased or decreased insulin signaling may be associated with term of infusions. In short-term lipid infusion, hyperinsulinemia may stimulate activation of IRS-1 and up-regulates the activation of Akt, which promote a metabolic switch characterized by increased glycolysis while impairing mitochondrial fatty acid oxidation. Over time, with persistent nutrient overload, cardiomyocytes become desensitized to systemic hyperinsulinemia insulin-signaling pathways resulting in impaired Akt signaling via various mechanisms such as exacerbation of lipotoxicity.

A reduced level of cardiac eNOS in animal models of obesity is reported to be a powerful predictor of coronary heart disease, because there is a significant relationship between the regulation of coronary blood flow and NO, which is dependent on activation and expression of eNOS [29, 30]. Recent studies have indicated that in endothelial cells, NO production was impaired, and phosphorylation of eNOS protein and eNOS mRNA expression and activity significantly decreased when FFA was elevated in the FFA group [16, 31]. Additionally, one of the AMPK targets that may be important in the cardiovascular system is eNOS [32]. Lipid infusion can reduce both phosphorylation of AMPK and eNOS and impair endothelial function [19]. AMPK was suggested as a signaling molecule involved in the pathophysiology of a variety of cardiac diseases and models. Recent studies have indicated that phosphorylation of AMPK was markedly reduced in the heart following high-fat diet-induced obesity in mice [15, 33], and total AMPK protein levels and phosphorylation of AMPK were dramatically reduced in cardiac tissue after intralipid plus heparin infusion for 5 h [15]. In the current study, we found that total AMPK, p-AMPK, and eNOS protein levels gradually but markedly deceased compared with the control group over time. Taken together, these data support the role of high FFA in the regulation of cardiovascular AMPK and eNOS.

## Conclusions

We found that plasma FFA levels, which were elevated using intralipid plus heparin infusion, impaired cardiac structure and function. Short-term lipid infusion increased, whereas long-term infusion decreased, PI3K–Akt–GLUT4 signaling in myocardium. Lipid infusion also inhibited AMPK–eNOS signaling in myocardium. We suggest that high FFA induced by lipid infusion plays an important role in the development of myocardial insulin resistance and cardiac dysfunction via altering PI3K–Akt-–GLUT4 and decreasing AMPK–eNOS signaling networks in the heart.

## Abbreviations

+dP/dt max: Maximum velocity of myocardial contraction
A: Peak late flow velocities
A’: Late diastolic peak velocities
Akt: Serine/threonine kinase
AMPK: Adenosine 5′-monophosphate -activated protein kinase
−dP/dt max: Maximum velocity of myocardial diastole
E: Peak early flow velocities
E’: Early diastolic peak velocities
EF %: Ejection fraction
eNOS: Endothelial nitric oxide synthase
FFA: Free fatty acids
FS %: Fractional shortening
GLUT4: Glucose transporter type 4
IRS-1: Insulin receptor substrate 1
IVSd: Interventricular septum thickness
LV: Left ventricular
LVIDd: LV inner dimension-diastole
LVIDs: LV inner dimension-systole
LYPWT: Left ventricular end-diastolic posterior wall thickness
NO: Nitric oxide
PI3K: Phosphatidylinositol 3-kinase
